# pyAudioAnalysis: An Open-Source Python Library for Audio Signal Analysis

**DOI:** 10.1371/journal.pone.0144610

**Published:** 2015-12-11

**Authors:** Theodoros Giannakopoulos

**Affiliations:** Computational Intelligence Laboratory, Institute of Informatics and Telecommunications, NCSR Demokritos, Patriarchou Grigoriou and Neapoleos St, Aghia Paraskevi, Athens, 15310, Greece; University of Pavia, ITALY

## Abstract

Audio information plays a rather important role in the increasing digital content that is available today, resulting in a need for methodologies that automatically analyze such content: audio event recognition for home automations and surveillance systems, speech recognition, music information retrieval, multimodal analysis (e.g. audio-visual analysis of online videos for content-based recommendation), etc. This paper presents pyAudioAnalysis, an open-source Python library that provides a wide range of audio analysis procedures including: feature extraction, classification of audio signals, supervised and unsupervised segmentation and content visualization. pyAudioAnalysis is licensed under the Apache License and is available at GitHub (https://github.com/tyiannak/pyAudioAnalysis/). Here we present the theoretical background behind the wide range of the implemented methodologies, along with evaluation metrics for some of the methods. pyAudioAnalysis has been already used in several audio analysis research applications: smart-home functionalities through audio event detection, speech emotion recognition, depression classification based on audio-visual features, music segmentation, multimodal content-based movie recommendation and health applications (e.g. monitoring eating habits). The feedback provided from all these particular audio applications has led to practical enhancement of the library.

## Introduction

The increasing availability of audio content, through a vast distribution of channels, has resulted in the need for systems that are capable of automatically analyzing this content. Depending on the individual types of distribution channels, the types of audio classes (speech, music, etc), the existence of other media (e.g. visual information) and the application-specific requirements, a wide range of different applications have emerged during the last years: music information retrieval, audio event detection, speech and speaker analysis, speech emotion recognition, multmodal analysis, etc. The purpose of the pyAudioAnalysis library is to provide a wide range of audio analysis functionalities through an easy-to-use and comprehensive programming design.

pyAudioAnalysis can be used to extract audio features, train and apply audio classifiers, segment an audio stream using supervised or unsupervised methodologies and visualize content relationships. The library is written in Python, which is a high-level programming language that has been attracting increasing interest, especially in the academic and scientific community during the past few years. Python is rather attractive for computational signal analysis applications mainly due to the fact that it provides an optimal balance of high-level and low-level programming features: less coding without an important computational burden. The initial problem of high computational demands is partly solved by the application of optimization procedures on higher level objects. In addition, compared to Matlab or other similar solutions, Python is free and can lead to standalone applications without the requirement of huge preinstalled binaries and virtual environments. Another great advantage of Python is that there exists an impressive number of libraries that provide functionalities related to scientific programming. Table 1 presents a list of related audio analysis libraries implemented in Python, C/C++ and Matlab.

**Table 1:** Related Work.

Name	Description
Yaafe	A Python library for audio feature extraction and basic audio I/O (http://yaafe.sourceforge.net/)
Essentia	An open-source C++ library for audio analysis and music information retrieval. Mostly focuses on audio feature extraction, basic I/O, while it also provides some basic classification functionalities http://essentia.upf.edu/
aubio	A C library for basic audio analysis: pitch tracking, onset detection, extraction of MFCCs, beat and meter tracking, etc. Provides wrappers for Python. http://aubio.org/
CLAM (C++ Library for Audio and Music)	A framework for research / development in the audio and music domain. Provides the means to perform complex audio signal analysis, transformations and synthesis. Also provides a graphical tool. http://clam-project.org/
Matlab Audio Analysis Library	A Matlab library for audio feature extraction, classification, segmentation and music information retrieval http://www.mathworks.com/matlabcentral/fileexchange/45831-matlab-audio-analysis-library. Can be used as companion matetrial for the book [1]
librosa	A Python library that implements some audio features (MFCCs, chroma and beat-related features), sound decomposition to harmonic and percussive components, audio effects (pitch shifting, etc) and some basic communication with machine learning components (e.g. clustering) https://github.com/bmcfee/librosa/
PyCASP	This Python library focuses on providing a collection of specializers towards automatic mapping of computations onto parallel processing units (either GPUs or multicore CPUs). These computations are presented through a couple of audio-related examples.
seewave	This is an R package for basic sound analysis and synthesis. Mostly focusing on feature extraction and basic I/O. https://cran.r-project.org/web/packages/seewave/index.html
bob	An open general signal processing and machine learning library (C++ and Python). http://idiap.github.io/bob/

A list of related libraries and packages focusing on audio analysis.

**Fig 1:**
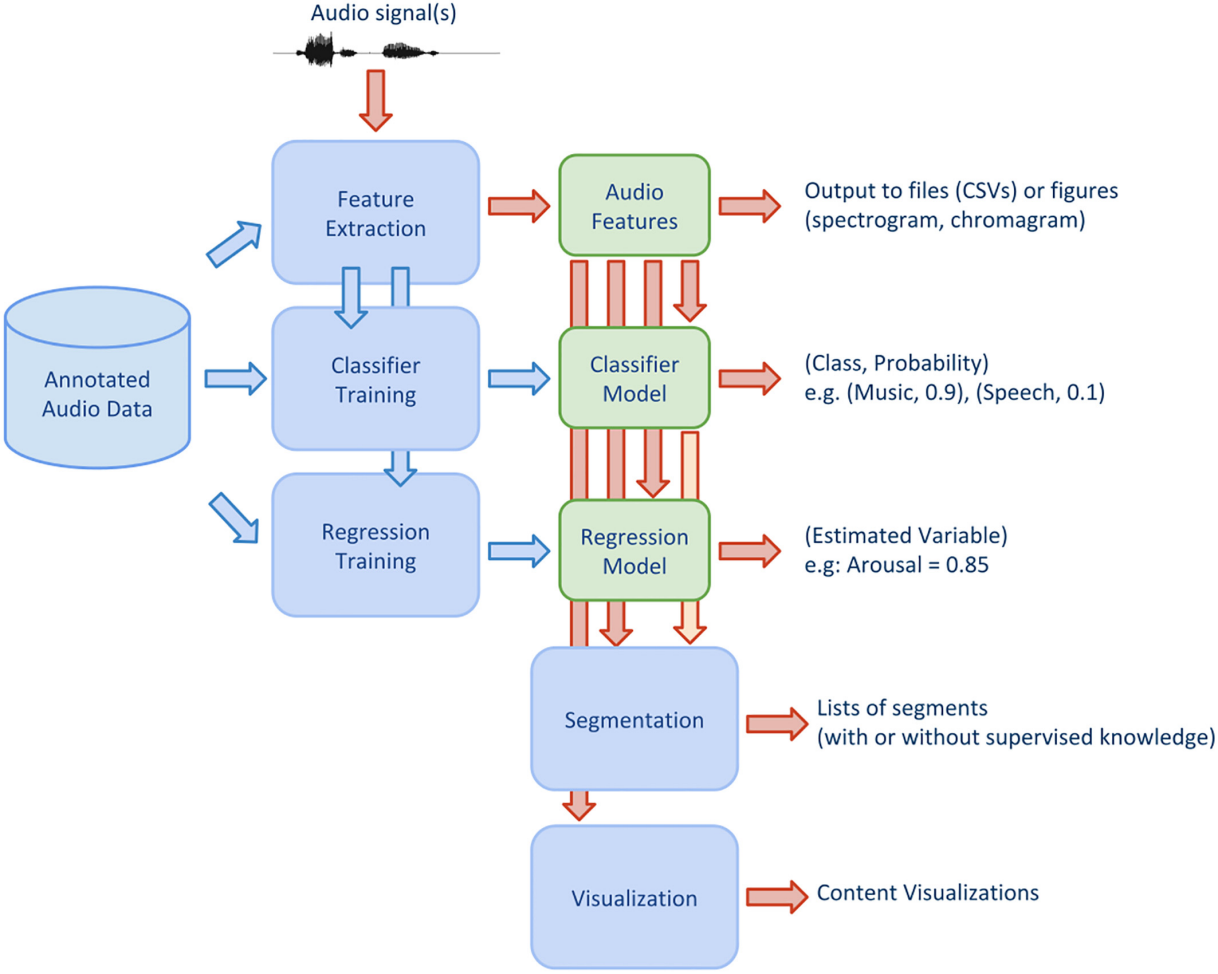
Library General Diagram.

**Fig 2:**
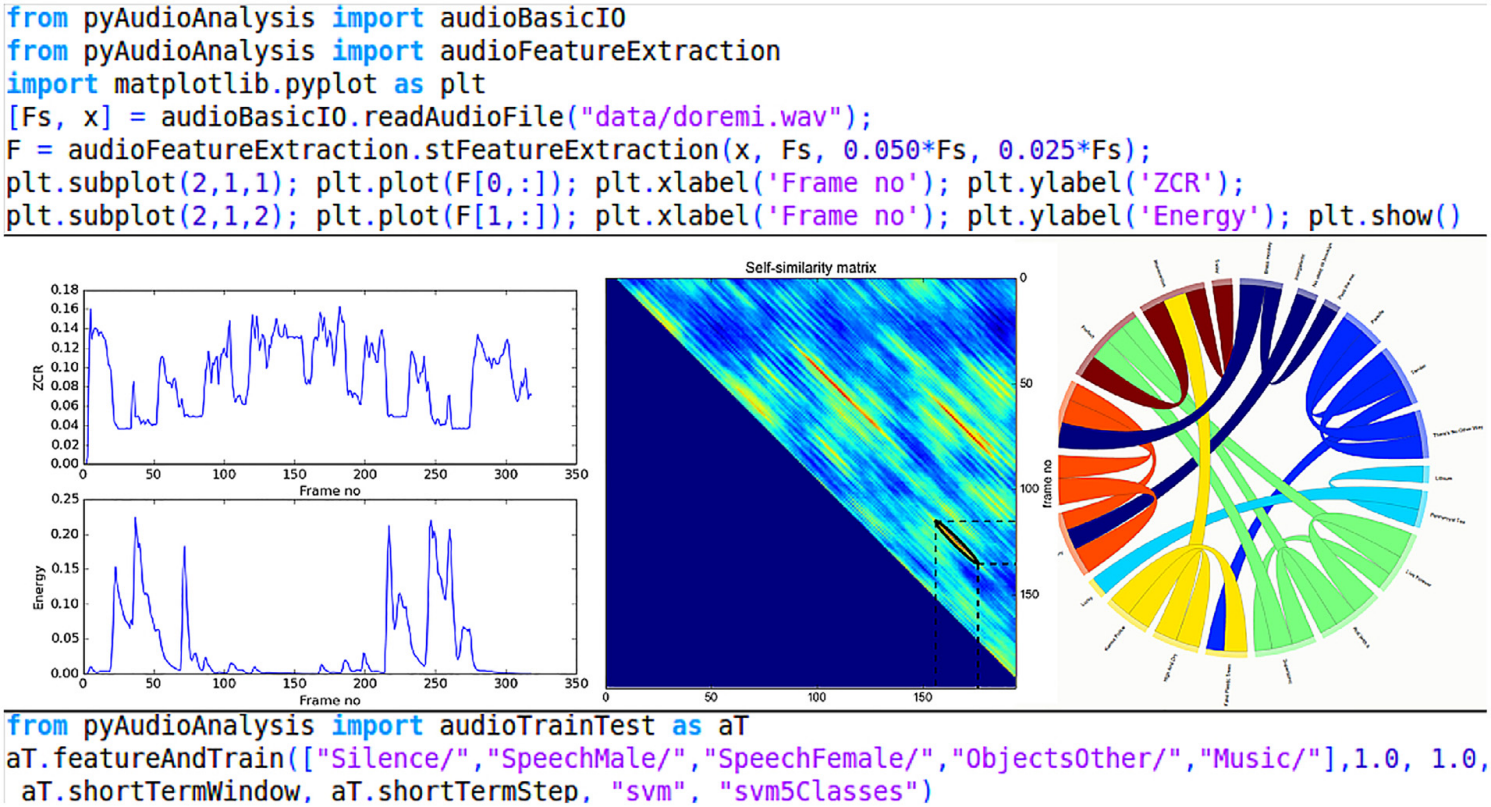
pyAudioAnalysis provides easy-to-use and high-level Python wrappers for several audio analysis tasks.

Fig 1 illustrates a conceptual diagram of the library, while Fig 2 shows some screenshots from the library’s usage. pyAudioAnalysis implements the following functionalities:

- Feature extraction: several audio features both from the time and frequency domain are implemented in the library.
- Classification: supervised knowledge (i.e. annotated recordings) is used to train classifiers. A cross-validation procedure is also implemented in order to estimate the optimal classifier parameter (e.g. the cost parameter in Support Vector Machines or the number of nearest neighbors used in the kNN classifier). The output of this functionality is a classifier model which can be stored in a file. In addition, wrappers that classify an unknown audio file (or a set of audio files) are also provided in that context.
- Regression: models that map audio features to real-valued variables can also be trained in a supervised context. Again, cross validation is implemented to estimate the best parameters of the regression models.
- Segmentation: the following supervised or unsupervised segmentation tasks are implemented in the library: fix-sized segmentation and classification, silence removal, speaker diarization and audio thumbnailing. When required, trained models are used to classify audio segments to predefined classes, or to estimate one or more learned variables (regression).
- Visualization: given a collection of audio recordings pyAudioAnalysis can be used to extract visualizations of content relationships between these recordings.

pyAudioAnalysis provides the following characteristics which, combined as a whole, are unique compared to other related libraries:

- General feature extraction and machine learning conceptual components are linked to form complete audio classification and segmentation solutions.
- Both state-of-the-art and baseline techniques are implemented to solve widely used audio analysis tasks.
- Pre-trained models are also provided for some supervised tasks (e.g. speech-music classification, music genre classification and movie event detection).
- All provided functionalities are written using distinct and simple code so that the conceptual algorithmic steps can be clearly presented in the context of an educational process.

## Feature Extraction

### Features description

This Section gives a brief description of the implemented features. For detailed description the reader can refer to the related bibliography [1–3]. The complete list of extracted features in pyAudioAnalysis is presented in Table 2. The time-domain features (features 1–3) are directly extracted from the raw signal samples. The frequency-domain features (features 4–34, apart from the MFCCs) are based on the magnitude of the Discrete Fourier Transform (DFT). Finally, the cepstral domain (e.g. used by the MFCCs) results after applying the Inverse DFT on the logarithmic spectrum.

**Table 2:** Audio Features.

Index	Name	Description
1	Zero Crossing Rate	The rate of sign-changes of the signal during the duration of a particular frame.
2	Energy	The sum of squares of the signal values, normalized by the respective frame length.
3	Entropy of Energy	The entropy of sub-frames’ normalized energies. It can be interpreted as a measure of abrupt changes.
4	Spectral Centroid	The center of gravity of the spectrum.
5	Spectral Spread	The second central moment of the spectrum.
6	Spectral Entropy	Entropy of the normalized spectral energies for a set of sub-frames.
7	Spectral Flux	The squared difference between the normalized magnitudes of the spectra of the two successive frames.
8	Spectral Rolloff	The frequency below which 90% of the magnitude distribution of the spectrum is concentrated.
9–21	MFCCs	Mel Frequency Cepstral Coefficients form a cepstral representation where the frequency bands are not linear but distributed according to the mel-scale.
22–33	Chroma Vector	A 12-element representation of the spectral energy where the bins represent the 12 equal-tempered pitch classes of western-type music (semitone spacing).
34	Chroma Deviation	The standard deviation of the 12 chroma coefficients.

Complete list of implemented audio features. Each short-term window is represented by a feature vector of 34 features listed in the Table.

### Short and mid-term analysis

The aforementioned list of features can be extracted in a short-term basis: the audio signal is first divided into short-term windows (frames) and for each frame all 34 features are calculated. This results in a sequence of short-term feature vectors of 34 elements each. Widely accepted short-term window sizes are 20 to 100 ms. In pyAudioAnalysis, the short-term process can be conducted either using overlaping (frame step is shorter than the frame length) or non-overlaping (frame step is equal to the frame length) framing.

Another common technique in audio analysis is the processing of the feature sequence on a mid-term basis, according to which the audio signal is first divided into mid-term windows (segments), which can be either overlaping or non-overlaping. For each segment, the short-term processing stage is carried out and the feature sequence from each mid-term segment, is used for computing feature statistics (e.g. the average value of the ZCR). Therefore, each mid-term segment is represented by a set of statistics. Typical values of the mid-term segment size can be 1 to 10 seconds. In cases of long recordings (e.g. music tracks) a long-term averaging of the mid-term features can be applied so that the whole signal is represented by an average vector of mid-term statistics.

### Tempo-related features

**Fig 3:**
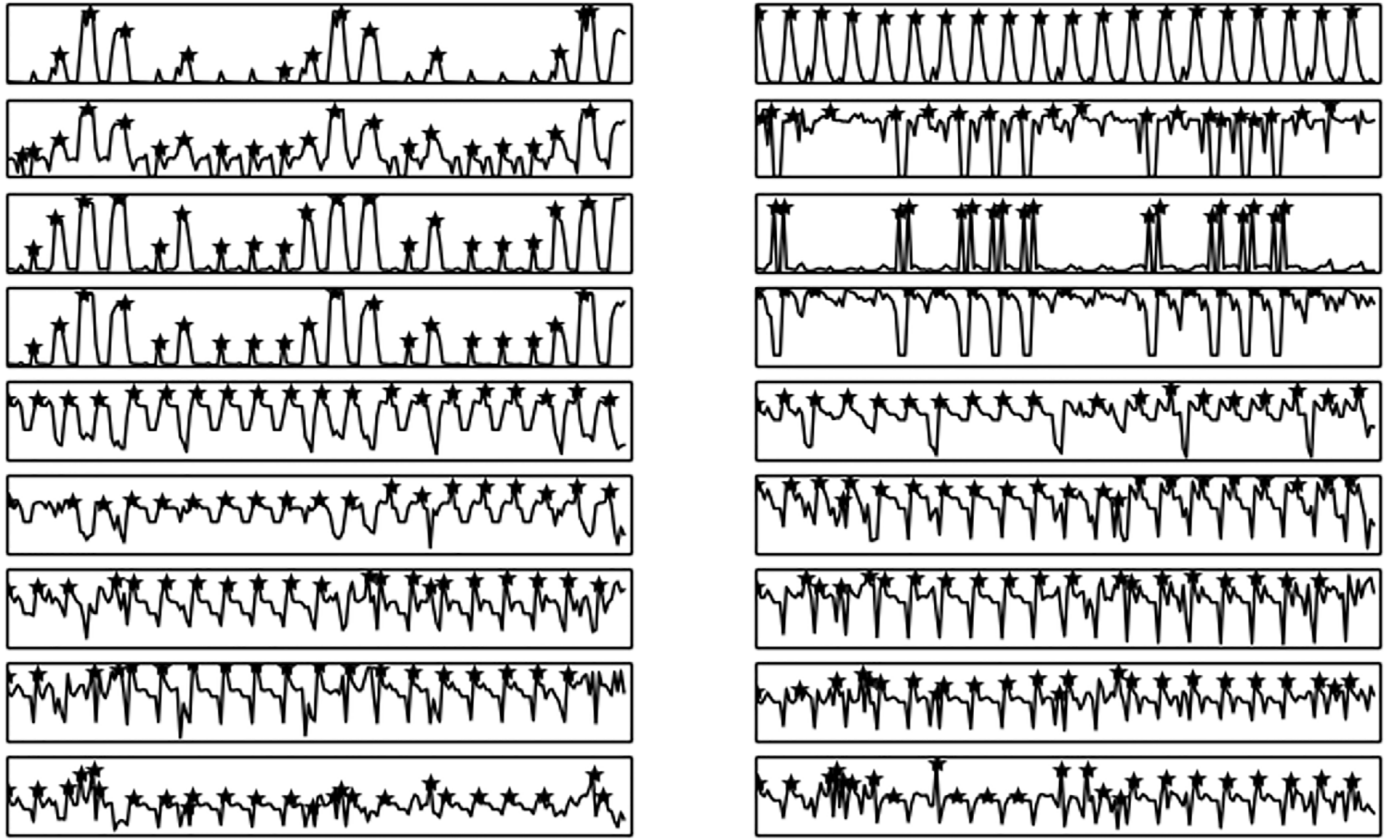
Local maxima detection for beat extraction. An example of local maxima detection on each of the adopted short-term features. The time distances between successive local maxima are used in the beat extraction process.

**Fig 4:**
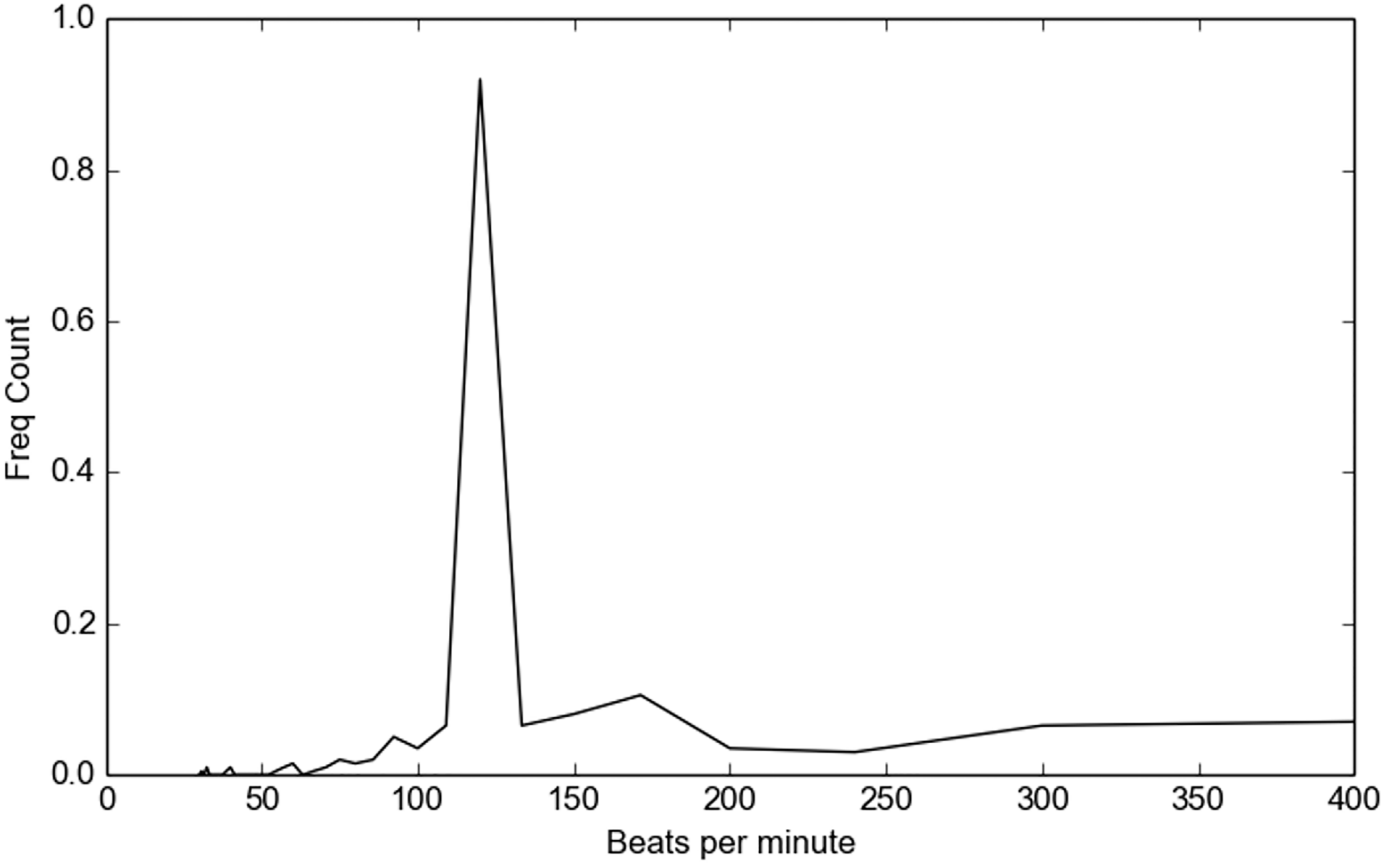
Beat histogram example. An aggregated histogram of time distances between successive feature local maxima. The histogram’s maximum position is used to estimate the BPM rate.

Automatic beat induction, i.e. the task of determining the rate of musical beats in time is a rather important task, especially for the case of music information retrieval applications [4, 5]. In this library, a straightforward approach for tempo calculation has been implemented. It adopts a local maxima detection procedure (see Fig 3), applied on a set of short-term feature sequences. An aggregated histogram (see Fig 4) of the time distances between successive local maxima is also computed and its maximum element corresponds to the most dominant time distance between successive beats. Finally, this detected value is used to compute the BPM rate. Apart from the BPM value itself, the ratio of the maximum histogram value by the total sum of histogram values is used as a feature, corresponding to the overall “dominance” of the detected beat rate.

It has to be noted that the two tempo-related features are only extracted for whole audio recordings (they are not computed in a short-term basis). Therefore, they can only be added to long-term averages of the mid-term features described in the previous section. For segment-classifiers of general audio classes (e.g. music vs speech) tempo features are not applicable.

## Audio Classification

Classification is probably the most important problem in machine learning applications. It refers to the task of classifying an unknown sample (in our case audio signal) to a set of predefined classes, according to some trained supervised model. The library provides functionalities for the training of supervised models that classify either segments or whole audio recordings. Support vector machines and the k-Nearest Neighbor classifier have been adopted towards this end. In addition, a cross-validation procedure is provided in order to extract the classifier with optimized parameters. In particular, the precision and recall rates, along with the F1 measure are extracted per audio class. Parameter selection is performed based on the best average F1 measure.

High-level wrapper functions are provided so that the feature extraction process is also embedded in the classification procedure. In this way, the users can directly classify unknown audio files or even groups of audio files stored in particular paths.

## Audio Regression

Regression is the task of estimating the value of an unknown variable (instead of distinct class labels), given a respective feature vector. It can also be rather important in the context of an audio analysis application, in cases there are mappings from audio features to a real-valued variable. A typical example is speech emotion estimation, where the emotions are not represented by discrete classes (e.g. Anger, Happiness, etc) but by dimensional representations (e.g. Valence—Arousal). The library supports SVM regression training in order to map audio features to one or more supervised variables. In order to train an audio regression model the user should provide a series of audio segments stored in separate files in the same folder. In addition, the user must provide a comma-separated-file (CSV), where the respective ground-truth values of the output variable are stored. During the training phase, for each CSV file a separate variable is trained.

Note that the regression training functionality also contains a parameter tuning procedure, where a cross-validation evaluation is performed, similar to that of the classification case. However, the performance measure maximized in the context of the regression training procedure is the Mean Square Error (MSE). In addition to that, for each tested parameter value, the MSE of the training data is also calculated to provide a measure of “overfitting”. Finally, the parameter tuning procedure returns the MSE of the “average estimator”, i.e. a method that always returns the average value of the estimated parameter (based on the training data), in order to provide a baseline performance measure. This is equivalent to the “random classifier” used as a worst-case performance when evaluating classification methods.

## Audio Segmentation

Audio segmentation focuses on splitting an uninterrupted audio signal into segments of homogeneous content. The term “homogeneous” can be defined in many different ways, therefore there exists an inherent difficulty in providing a global definition for the concept. The library provides algorithmic solutions for two general subcategories of audio segmentation:

- the first contains algorithms that adopt some type of “prior” knowledge, e.g. a pre-trained classification scheme. For that type of segmentation the library provides a fix-sized joint segmentation—classification approach and an HMM-based method.
- the second type of segmentation is either unsupervised or semi-supervised. In both cases, no prior knowledge on the involved classes of audio content is used. Typical examples are silence removal, speaker diarization and audio thumbnailing.

### Supervised audio segmentation

#### Fix-sized segmentation

**Fig 5:**
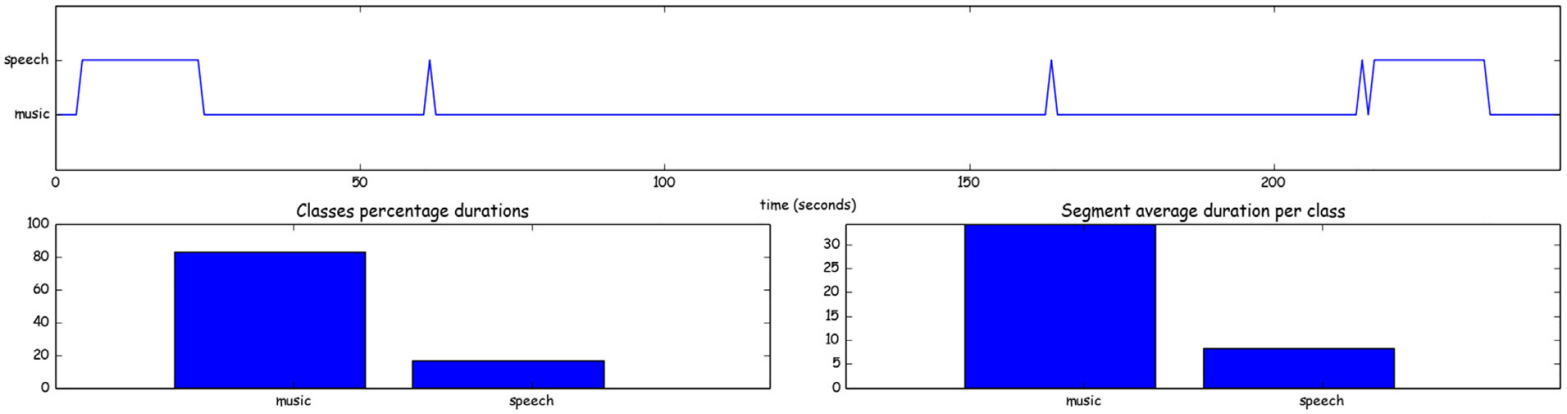
Segmentation Example. Supervised segmentation results and statistics for a radio recording. A binary speech vs music classifier is used to classify each fix-sized segment.

This straightforward way of segmenting an audio recording to homogeneous segments splits the audio stream into fixed-size segments and classifies each segment separately using some supervised model. Successive segments that share a common class label are merged in a post-processing stage. In addition, the library extracts some basic statisics (e.g. Fig 5).

#### HMM-based segmentation

Hidden Markov Models (HMMs) are stochastic automatons that follow transitions among states based on probabilistic rules. When the HMM arrives at a state, it emits an observation, which in the case of signal analysis is usually a continuous feature vector. HMMs can be used for recognizing sequential labels based on a respective sequence of audio feature vectors. This is achieved through finding the sequence of states that emits a particular sequence of observations with the highest probability. The answer to this question is given by a dynamic programming methodology, the *Viterbi* algorithm [1, 2, 6].

pyAudioAnalysis provides the ability to train and test HMMs for joint audio segmentation-classification. In order to train the HMM model, the user has to provide annotated data in a rather simple comma-separated format that includes three columns: segment starting point, segment ending point and segment label. One annotation file must be provided for each respective audio recording. The set of audio files and respective annotation files forms the training set.

Table 3 presents the performance results of the implemented joint segmentation-classification approaches. Towards this end a dataset of annotated radio broadcast recordings has been used, similar to the one used in [7], in the context of speech-music discrimination. This dataset is composed of more than 50 recordings of 10 hours total duration. The classification task is binary (speech vs music). Results indicate that the HMM segmentation-classification procedure outperforms the fix-sized approach by almost 2% and 1% for the kNN and the SVM classifiers respectively.

**Table 3:** HMM joint segmentation classification performance.

Method	Accuracy
Fix-sized window kNN	93.1%
Fix-sized window SVM	94.6%
HMM	95.1%

Average accuracy of the of each segmentation-classification method on a radio broadcasting dataset.

### Unsupervised audio segmentation

#### Silence removal

A semi-supervised silence removal functionality is also provided in the library. The respective function takes an uninterrupted audio recording as input and returns segment endpoints that correspond to individual audio events, removing “silent” areas of the recording. This is achieved through a semi-supervised approach which performs the following steps:

- The short-term features of the whole recording are extracted
- An SVM model is trained to distinguish between high-energy and low-energy short-term frames. In particular, 10% of the highest energy frames along with the 10% of the lowest are used to train the SVM model
- The SVM classifier is applied (with a probabilistic output) on the whole recording, resulting in a sequence of probabilities that correspond to a level of confidence that the respective short-term frames belong to an audio event (and do not belong to a silent segment).
- A dynamic thresholding is used to detect the active segments.

**Fig 6:**
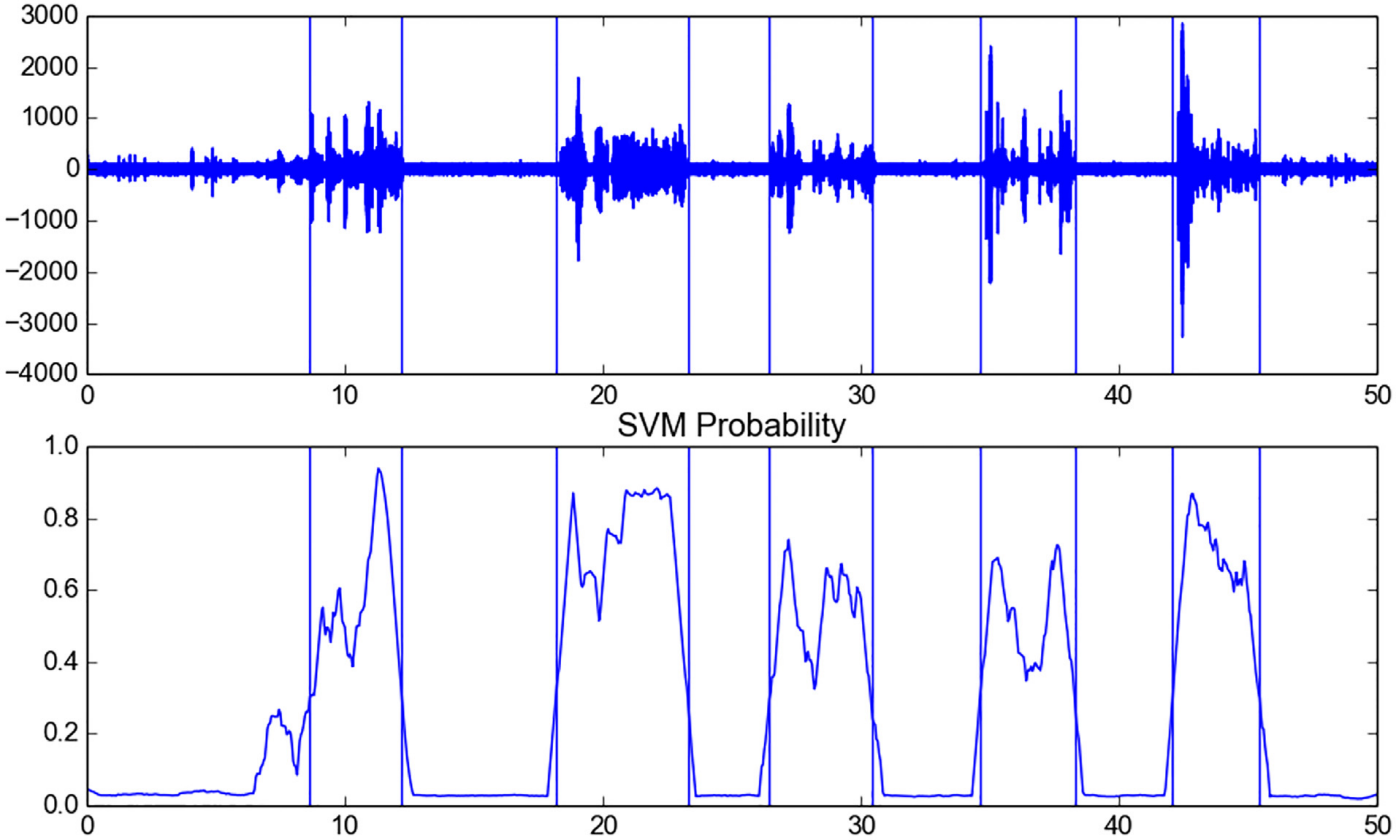
Silence Removal Example. An example of applying the silence removal method on an audio recording. Upper subfigure represents the audio signal, while the second subfigure shows the SVM probabilistic sequence.

Fig 6 shows an example of the silence removal method.

#### Speaker Diarization

Speaker diarization is the task of automatically answering the question “who spoke when”, given a speech recording [8, 9]. Extracting such information can help in the context of several audio analysis tasks, such as audio summarization, speaker recognition and speaker-based retrieval of audio.

Speaker diarization is usually treated as a joint segmentation—clustering processing step, where speech segments are grouped into speaker-specific clusters. This straightforward and mainstream methodology is implemented in pyAudioAnalysis as a baseline speaker diarization method, along with a two-step smoothing approach (see details below). However, it has been proven that the choice of the feature space to represent speech is fundamental to the success of most diarization steps. To produce a more feature—independent result, we have also selected to implement a version of the Fisher Linear Semi-Discriminant analysis (FLsD) method proposed in [10], which finds a near-optimal feature subspace in terms of speaker discrimination. This method is completely unsupervised as it leverages information from the sequential structure of the speech signals. The particular steps of the speaker diarization method adopted in pyAudioAnalysis are listed below:

- *Feature extraction* Extract MFCCs in a short-term basis and means and standard deviation of these feature sequences on a mid-term basis, as described in the Feature Extraction stage. Proposed short-term window size is 50 ms and step 25 ms, while the size of the texture window (mid-term window) is 2 seconds with a 90% overlap (i.e. mid-term step is 0.2 seconds). In addition, pyAudioAnalysis implements the ability to add supervised knowledge during the feature extraction stage. By “supervised” we do not refer to the speakers of the analyzed recording but to a set of predefined speakers model. For example, the mid-term feature statistics vector is enhanced by adding the probabilities that the respective audio segments belongs to a male or female speaker, where this gender classification model has been trained on a set of annotated segments beforehand.
- *(Optional) FLsD step* In this stage we obtain the near-optimal speaker discriminative projections of the mid-term feature statistic vectors using the FLsD approach. In particular, each fixed-size texture segment (2 sec) is assigned a new *speaker thread* and the feature vectors within this segment are used to obtain the speaker-thread mean feature vector and scatter matrix and also to update the overall within-class thread and mixed-class scatter matrices used in the FLsD method. At the end, the scatter matrices are given as arguments to the Fisher criterion to obtain the optimal speaker-discriminative subspace.
- *Clustering* A k-means clustering method is performed (either on the original feature space or the FLsD subspace). This yields a sequence of cluster labels (one label per texture window). The k-means algorithm takes as argument a user-provided number of clusters (speakers). In case that this is not a-priori known, the clustering process is repeated for a range of number of speakers and the Silhouette width criterion [11] is used to decide about the quality of the clustering result in each case and therefore the optimal number of speakers.
- *Smoothing* A two-step smoothing process is applied combining (a) a median filtering on the extracted cluster IDs and (b) a Viterbi Smoothing step.

Table 4 presents an evaluation of the implemented speaker diarization methods on a subset of the widely used Canal9 dataset [12]. As performance measures, the average cluster purity (ACP) and the average speaker purity (ASP) have been adopted, along with their harmonic mean (F1 measure).

**Table 4:** HMM joint segmentation classification performance.

FLsD	StWin (ms)	ACP	ASP	F1
No	MFCCs (mean)	77	72.5	74.5
Yes	MFCCs (mean)	83	80	81.5
No	MFCCs (mean), Gender	73	70	71.5
Yes	MFCCs (mean), Gender	83	81	82
No	MFCCs (mean-std)	76	72	74
Yes	MFCCs (mean-std)	84	82	83
No	MFCCs (mean-std), Gender	78	73	75.5
Yes	MFCCs (mean-std), Gender	83	81	82
No	MFCCs (mean-std), Spectral	70	63	66.5
Yes	MFCCs (mean-std), Spectral	85	81	83
No	MFCCs (mean-std), Spectral, Gender	68	61	64.5
Yes	MFCCs (mean-std), Spectral, Gender	85	81	83

Performance measures of the implemented speaker diarization method for different initial feature sets. The FLsD method provides a more robust behavior independently from the initial feature space, since it helps to discover a speaker-discriminant subspace.

These results prove that the FLsD approach achieves a performance boosting, related to the respective initial feature space. In addition, it is rather important to emphasize that the performance of the FLsD is robust and independent to the initial feature representation: the overall performance of the FLsD method ranges from 81% to 83%, despite the fact when the diarization method is applied directly on the initial feature space the performance varies from 61% to 78%. This proves that the *FLsD approach manages to discover a speaker-discriminant subspace*.

#### Audio Thumbnailing

**Fig 7:**
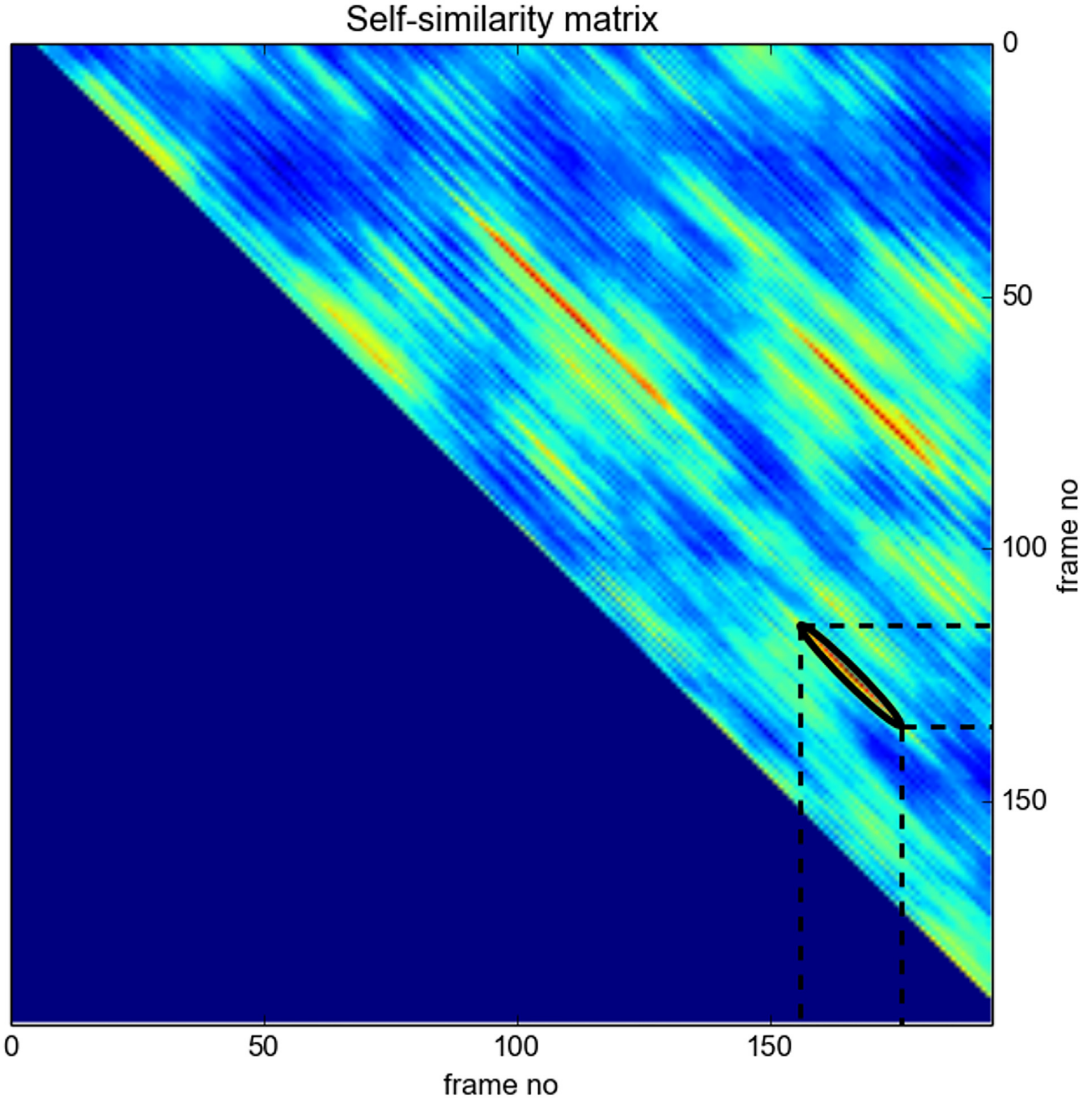
Audio thumbnailing example. Example of a self-similarity matrix for the song “Charmless Man” by Blur. The detected diagonal segment defines the two thumbnails, i.e. segments (115.0sec–135.0sec) and (156.0sec–176.0sec).

This is the task of extracting the most representative part of a music recording, which, in popular music, is usually the chorus. The library actually implements a variant of the method proposed in [13], which is based on finding the maximum of a filtered version of the self-similarity matrix. The results (i.e. the extracted thumbnails) are exported to respective audio files, while the self-similarity (along with the detected areas) can also be visualized (see Fig 7).

## Audio Visualization

**Fig 8:**
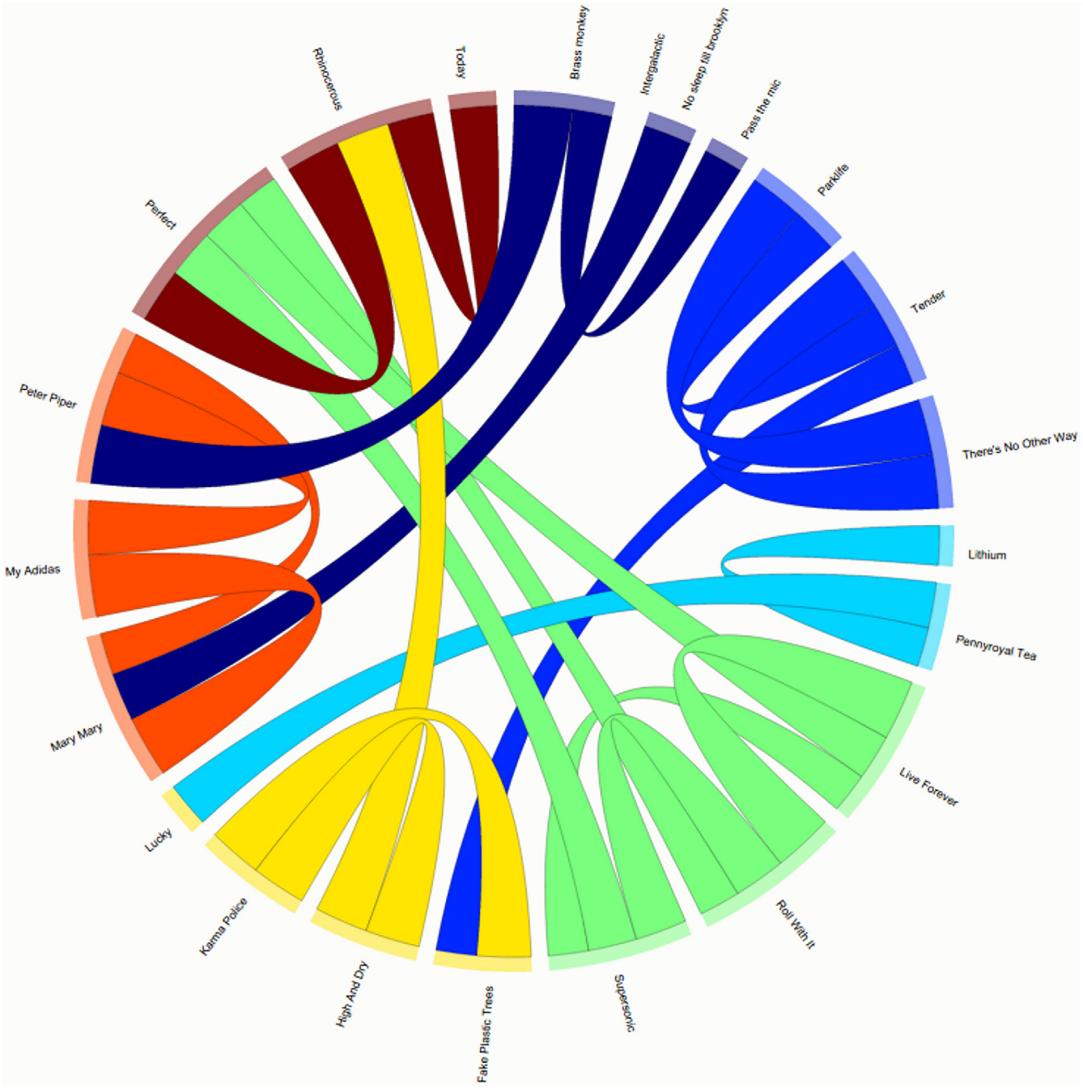
Chordial Content Visualization Example. Different colors of the edges and nodes (recordings) represent different categories (artists in our case).

Content-based visualization is rather important in many types of multimedia indexing, browsing and recommendation applications, while it can also be used to extract meaningful conclusions regarding the content relationships. In pyAudioAnalysis, a method that visualizes content similarities between audio signals is provided. The adopted methodology is the following:

- Given: a set of audio files (e.g. stored in WAV files stored in a particular path). Prior and manual categorization information can also be provided through the filenames theirselves: if the filename format: <category name> --- <sample name> is provided, then the input signals are supposed to be classified to the given categories. This information is either used only for visualization or for supervised dimensionality reduction. For example, the filename Blur --- Charmless Man.wav assigns the category label “Blur” to the signal. Such information can also be driven from MP3 tag information: for example pyAudioAnalysis provides an MP3-to-WAV conversion functionality that produces files with the aforementioned filename format, where the category name is taken from the “artist” MP3 tag.
- Extract mid-term features and long-term averages in order to produce one feature vector per audio signal.
- The feature representation can be (optionally) projected to a lower dimension. Towards this end, either Principal Component Analysis (PCA) or Linear Discriminant Analysis (LDA) is used. PCA is unsupervised, however LDA requires some type of supervised information. If available, this infomartion is stemming from the aforementioned “category” label.
- A similarity matrix is computed based on the cosine distances of the individual feature vectors.
- The similarity matrix is used to extract a chordial representation (Fig 8) that visualize the content similarities between the audio recordings. Towards this end, d3js is used (http://d3js.org/). d3js is a JavaScript library for manipulating and visualizing documents based on data.

## Implementation Issues

### Dependencies

pyAudioAnalysis is written in Python v2.7 using widely used open libraries. The most important of these external dependencies are the following:

- Numpy (http://www.numpy.org/) is a fundamental library for numeric computations using Python. It has been mainly used for it’s arrays and matrices representation and handling, along with a set of respective basic array functionalities.
- Matplotlib (http://matplotlib.org/) offers a 2D plotting functionality, similar to that of MATLAB.
- SciPy (http://www.scipy.org/) is the core of the SciPy Python-based ecosystem that provides optimized numeric computations and scientific routines. pyAudioAnalysis uses SciPy for basic signal processing procedures (e.g. convolution), linear computations, FFT computation and WAVE file IO.
- MLpy (http://mlpy.sourceforge.net/) is a Python package for Machine Learning and it has been mainly used for its kMeans and SVM implementation (SVM is actually a python wrapper of the LibSVM implementation).
- sklearn (http://scikit-learn.org/stable/) is another Machine Learning and Data Mining package and has been used for its LDA and HMM implementations.

### Computational Demands

Python, as a high-level programming language, introduces a high execution overhead (related to C for example), mainly due to its dynamic type functionalities and its interpreted execution. pyAudioAnalysis has managed to partly overcome this issue, mainly through taking advantage of the optimized vectorization functionalities provided by Numpy. Table 5 presents the computational demands on three different computers that cover a very wide range of computational power: (1) a standard modern workstation with a Intel Core i7-3770 CPU (4 cores at 3.40GHz) (2) a 2009 HP Pavilion dm3-1010 laptop with a Intel Pentium SU4100 Processor (2 cores at 1.3GHz) and (3) a Raspberry PI 2 with a 700 MHz single-core ARM1176JZF-S CPU. In particular, we present the computational ratios, i.e. the ratio of the total signal duration by the execution time. So, “115 x realtime” means that a 1-hour recording is processed by the respective procedure in almost 31 seconds. Most of the procedures are executed in high time performance ratios making them practical for real-world problems.

**Table 5:** Realtime ratios for some basic functionalities and different devices.

Procedure	Realtime Ratio 1	Realtime Ratio 2	Realtime Ratio 3
Short-term feature extraction	115	16	2.2
Mid-term segment classification	100	14	2
Fix-sized segmentation-classification	100	13	2
HMM-based segmentation-classification	100	13	2
Silence Removal	105	13	2
Audio Thumbnailing	450	16	7
Diarization—no FLsD	28	5	0.6
Diarization—FLsD	7	2	0.3

Realtime ratios express how many times faster than the actual signal’s duration is the respective computation. The particular values have been calculated for mono—16kHz signals. In addition, a 50 ms short-term window has been adopted. Both of these parameters (sampling rate and short-term window step) have a linear impact on the computational complexity of all functionalities. All of the functionalities are independent to the input signal’s duration apart from the audio thumbnailing and the diarization methods. For these methods, the particular ratios have been extracted using a 5-minute signal as input.

### Code organization

The library code is organized in six Python files. In particular:

- audioAnalysis.py: implements the command-line interface of the basic functionalities of the library, along with some recording functionalities.
- audioFeatureExtraction.py implements all feature extraction methods.
- audioTrainTest.py implements the audio classification prodecures.
- audioSegmentation.py implements audio segmentation functionalities, e.g. fixed-sized segment classification and segmentation, speaker diarization, etc.
- audioBasicIO.py includes some basic audio IO functionalities as well as file convertions
- audioVisualization.py produces user-friendly and representative content visualizations

All basic functionalities can be achieved in a command-line manner through audioAnalysis.py. Of course, the programmer can also use the individual files for including particular methods via coding. For example, training a classifier from code can be achieved as follows:

from pyAudioAnalysis import audioTrainTest as aT aT. featureAndTrain([“Classical/”, “Electronic/”, “Jazz/”], 1.0, 1.0, aT. shortTermWindow, aT. shortTermStep, “svm”, “svmMusicGenre3”, True)

or from command-line (via audioAnalysis.py) as follows:

python audioAnalysis.py trainClassifier -i Classical/ Electronic/ Jazz/ --method svm -o svmMusicGenre3

pyAudioAnalysis has a rather detailed wiki (https://github.com/tyiannak/pyAudioAnalysis/wiki) that shows how to call every single functionality described in this paper, either from command-line or via source code.

## Use-cases

pyAudioAnalysis has been directly used in several audio analysis applications. Some examples include:

- Content-based multimodal movie recommendation [14].
- Depression estimation [15].
- Speech emotion recognition [16].
- pyAudioAnalysis has been also used for evaluating features and segmentation approaches in the context of the BitBite startup (http://www.thebitbite.com/) which focuses on providing real-time methods for monitoring people’s eating habits.
- Estimating the quality of urban soundscape using audio analysis [17]. In this work, pyAudioAnalysis has been used to extract audio features, perform semi-supervised dimensionality reduction and to map these content representations to soundscape quality levels through regression.

## Conclusions

In this paper we have presented pyAudioAnalysis, an open-source Python library that implements a wide range of audio analysis functionalities and can be used in several applications. Using pyAudioAnalysis one can classify an unknown audio segment to a set of predefined classes, segment an audio recording and classify homogeneous segments, remove silence areas from a speech recording, estimate the emotion of a speech segment, extract audio thumbnails from a music track, etc. High-level wrappers and command-line usage are also provided so that non-programmers can achieve full functionality. The range of audio analysis functionalities implemented in the library covers most of the general audio analysis spectrum: classification, regression, segmentation, change detection, clustering and visualization through dimensionality reduction. Therefore pyAudioAnalysis can be used as a basis to most general audio analysis applications.

pyAudioAnalysis is kept being enhanced and new components are to be added in the near future. In particular, the main ongoing directions are: (a) implementation of an audio fingerprinting functionality to be adopted in the context of an audio retrieval system (b) optimize all feature extraction functionalities by accelerating the critical functions using NVIDIA GPUs parallelization through Cuda programming.
